# The “Genomic Code”: DNA Pervasively Moulds Chromatin Structures Leaving no Room for “Junk”

**DOI:** 10.3390/life11040342

**Published:** 2021-04-13

**Authors:** Giorgio Bernardi

**Affiliations:** 1 Science Department, Roma Tre University, Viale Marconi 446, 00146 Rome, Italy; gbernardi@uniroma3.it; Tel.: +39-33-540-5892; 2 Stazione Zoologica Anton Dohrn, Villa Comunale, 80121 Naples, Italy

**Keywords:** Genomic code, chromatin structure, junk DNA

## Abstract

The chromatin of the human genome was analyzed at three DNA size levels. At the first, compartment level, two “gene spaces” were found many years ago: A GC-rich, gene-rich “genome core” and a GC-poor, gene-poor “genome desert”, the former corresponding to open chromatin centrally located in the interphase nucleus, the latter to closed chromatin located peripherally. This bimodality was later confirmed and extended by the discoveries (1) of LADs, the Lamina-Associated Domains, and InterLADs; (2) of two “spatial compartments”, A and B, identified on the basis of chromatin interactions; and (3) of “forests and prairies” characterized by high and low CpG islands densities. Chromatin compartments were shown to be associated with the compositionally different, flat and single- or multi-peak DNA structures of the two, GC-poor and GC-rich, “super-families” of isochores. At the second, sub-compartment, level, chromatin corresponds to flat isochores and to isochore loops (due to compositional DNA gradients) that are susceptible to extrusion. Finally, at the short-sequence level, two sets of sequences, GC-poor and GC-rich, define two different nucleosome spacings, a short one and a long one. In conclusion, chromatin structures are moulded according to a “genomic code” by DNA sequences that pervade the genome and leave no room for “junk”.

## 1. Introduction

The term “genome” was coined one hundred years ago to define “the haploid chromosome ensemble together with the associated protoplasm”, by Hans Winkler [1], a Professor of Botany at the University of Hamburg (and a frequent visitor at the Stazione Zoologica of Naples; Christiane Groeben, personal comm.). This definition became popular only after the discoveries of three pillars of the human genome, the double helix [2], the regulatory sequences [3], and the genomic code [4]. While the double helix concerned the totality of the genome, coding and regulatory sequences occupied very small percentages of it, leaving vast spaces in the genome that were reasonably proposed to correspond to “junk DNA” [5]. Ohno identified this fraction of the genome to accommodate for large numbers of deleterious mutations that would otherwise result in heavy mutation load, if they were in functional regions of the genome [5]. In this study, however, we will argue that this portion of the genome is functional, and thus is not to be considered as junk, yet not coding for proteins, and as such can withstand mutations without altering their function.

This article (which was written during the COVID pandemic, when the University was closed) presents a new general picture of the organization of the human genome based on DNA sequences. It is focused on three major topics: (1) The old discovery of the bimodal compositional compartmentalization of the human genome and a comparison with later results from other approaches; (2) Recent investigations that link GC-poor and GC-rich DNA sequences characterized by compositionally flat and single- or multi-peak structures, respectively, to the long-range chromatin compartments, to the short-range chromatin sub-compartments corresponding to flat isochores and isochore loops, and to the short sequences responsible for nucleosome binding; and (3) The “genomic code”, the fourth pillar of the genome, which underlies the moulding of chromatin by DNA sequences; while the “genomic code” was discovered four years ago and discussed [6,7,8,9], a more complete picture will be presented here.

## 2. Isochores, Chromosomal Bands, Replication Timings, Evolutionary Transitions

**Figure 1:**
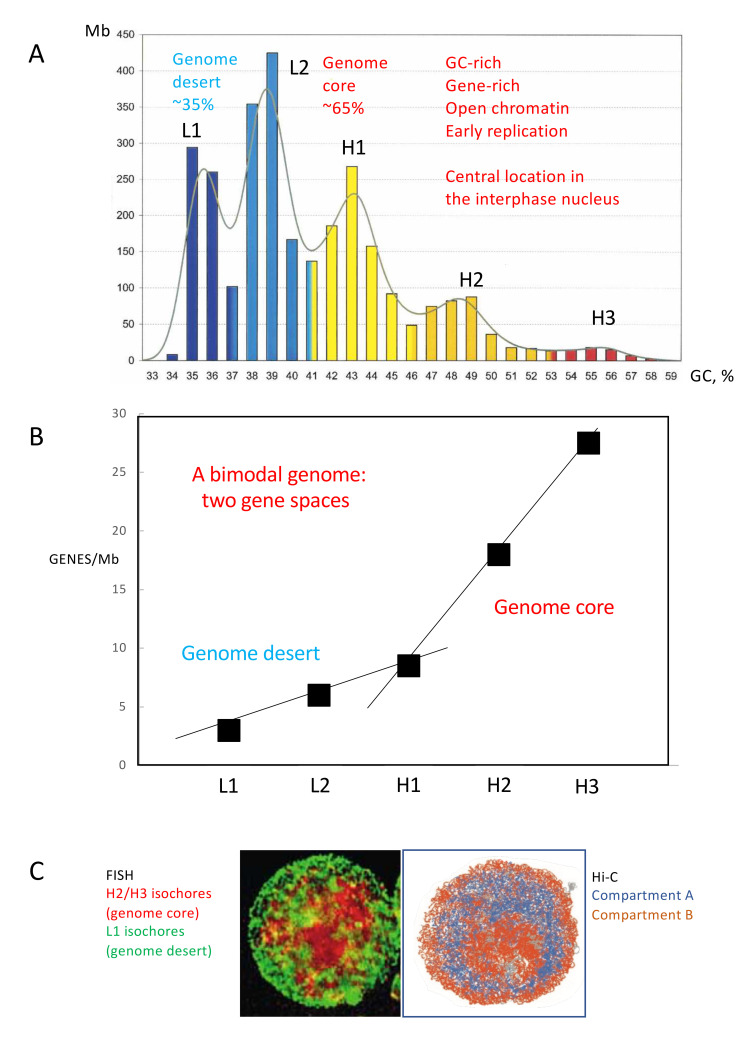
(**A**) Isochore families. The histogram displays the isochores from the human genome as pooled in bins of 1% GC. The Gaussian profile shows the distribution of isochore families, which are represented in different colours. Gene densities define a “genome desert”, comprising isochore families L1 and L2^−^, and a “genome core”, comprising isochore families L2^+^, H1, H2, H3; L2^−^ and L2^+^ sub-families are separated by a vertical broken red line. (Modified from [37]). (**B**) Gene distribution in the isochore families of the bovine genome. (Drawn from data of [37]); Two different lines of gene concentration characterized by different slopes were drawn through L1-H1 and H1-H3 points. (**C**) A FISH pattern of H2/H3 and L1 isochores (a clearer version of Figure 2A3 of [38]) is compared with a Hi-C pattern (modified from [39]), in which case compartment A is central, compartment B is peripheral and nucleolar.

The structural approach to the study of the genome, a top-down approach initiated sixty years ago [10], led to an important discovery: the demonstration of “major DNA components” in the mammalian genome [11], which, incidentally, occurred very close in time with the “junk DNA” proposal [5]. Indeed, (1) it disposed of the then generally accepted view that the DNA of higher organisms showed a continuous variation in GC level [12]; and (2) proposed that the genome of vertebrates is “a mosaic of isochores” [11,13,14,15], as we called the “major DNA components”, namely the “fairly homogeneous sequences” that belong, in the case of the human genome, in five families, L1, L2, H1, H2, H3, characterized by increasing GC levels (Figure 1A). The human genome was later estimated to comprise ~3200 isochores having an average size of 0.9 Mb [16]. More recently, (1) the human genome melting map was shown to co-vary very strongly with GC levels [17]; and (2) an approach, “isosegmenter”, was developed which allowed segmenting vertebrate genomes into isochores in a fast and completely automatic manner [18].

Three lines of evidence connect isochores with important genome structures and with genome evolution.

Our early proposal that the DNAs of Giemsa and Reverse bands correspond to GC-poor and GC-rich isochores, respectively [15], was followed by many investigations that defined the correlations between isochores and chromosomal bands from early prophase to metaphase [19,20,21,22].

Mammalian chromosomes were known to be replicated from many origins, adjacent starting points initiating replication at the same time, early and late in Reverse and Giemsa bands, respectively. More recent investigations confirmed the expected correlation between replication timing and isochore composition of the bands [23,24,25]. In fact, replicons located in a given isochore always show either all early or all late replication timings; moreover, early-replication isochores are short and GC-rich, late-replicating isochores long and GC-poor [21].

A third important point concerned the GC enrichment of isochores at the emergence of warm-blooded vertebrates. This stabilized thermodynamically at the higher body temperature of the latter a large part of the genome from cold-blooded vertebrates (a smaller part did not change its base composition) and was responsible for the compositional bimodal structure of the genome of warm-blooded vertebrates [26,27,28,29,30]. Moreover, it supported the idea that GC-rich isochores arose from adaptive evolution [19,29]. Interestingly, some fishes living at high temperatures showed GC-rich isochores that were absent in evolutionarily close species living at lower temperatures [26]. This GC-specific enrichment was also found in coding sequences of some turtles and crocodiles [31,32,33,34].

Finally, it should be mentioned that the initial sequencing and analysis of the human genome published by the International Human Genome Sequencing Consortium, IHGSC (2001) [35] comprised a number of misunderstandings about isochores that were discussed and clarified [36]. These problems seem now to be solved (see Section 11.4).

## 3. Sequence Distribution in the Human Genome and an Early View of the Genomic Code

The fractionation of the genome into families of isochores allowed localizing sequences in the genome. The first localizations were those of mammalian globin genes. These showed that while the GC-poor ß and γ globin genes were located in L2 isochores, the GC-rich α globin gene was located in H3 isochores, indicating that the GC levels of globin genes are correlated with the base composition of the isochores in which they are embedded [40]. The unavailability of additional gene probes at that time led us to concentrate on the genome localization (1) of two families of repeated sequences, LINEs and SINEs; and (2) of integrated viral sequences, since we had previously observed that the GC-rich bovine leukemia virus, BLV, was integrated in GC-rich H3 isochores [41].

The first approach led to the demonstration that GC-poor LINEs and GC-rich SINEs were mainly located in GC-poor isochores L1 and L2 [42] and GC-rich isochores H2 and H3 [43], respectively, another indication of a correlation between GC levels of specific sequences and GC levels of the “host” isochores. Since the two sets of repeats occupied vast regions, this also was an early evidence of a compositional bimodality in the human genome.

The second approach indicated that a compositionally matching integration was shown not only by BLV (as already mentioned), but also by all the GC-rich and GC-poor viral sequences tested. In fact, the viral sequences were expressed only when a compositionally matching integration was satisfied, namely when the situation was that of host genes [44,45].

Later on, an increasing number of protein-coding sequences were localized and indicated that linear compositional correlations were holding between exons (and their codon positions) and the isochores in which they were embedded [19,46,47], as well as between exons and the corresponding introns [26,48,49,50].

In fact, the correlations between coding and flanking non-coding sequences and those among the three codon positions led to a first definition of the “genomic code” [27,51], which stressed the fact that genomes are not random assemblies of coding and non-coding sequences, but systems obeying precise organization rules. This viewpoint was supported by: (1) the compositional compartmentalization of the human genome into isochores; (2) the localization of GC-poor/GC-rich isochores in Giemsa and Reverse bands, as well as their association with late and early replications; and (3) the evolutionary origin of GC-rich isochores and the bimodal structure of the genome of warm-blooded vertebrates (see the following section).

## 4. Gene Spaces

An important point concerning the distribution of coding sequences in the genome was its bimodality, as previously observed for GC-rich and GC-poor repeated sequences and for integrated viral sequences. Indeed, gene concentration was low and increased slowly with increasing GC in GC-poor isochores, whereas it increased rapidly in GC-rich isochores [29,36,45,46,47,52] reaching the highest level in the H3 isochore family, which was called the “genome core” [27,28], a definition later extended to all gene-rich isochores, including the H1 and H2 families. In fact, the break between the two slopes of gene concentration [47] defined two “gene spaces” [29], the GC-poor, gene-poor “empty quarter”, later called “genome desert” [45], and the GC-rich, gene-rich “genome core” already mentioned. As expected, the bimodality in protein-coding gene concentration could also be defined (see Figure 1B) using the 19,179 protein-coding genes of the bovine genome [37].

Another difference between the two gene spaces concerned the structures of the genes present in them. The first observation along this line concerned the GC-poor human dystrophin gene which was shown to be over 1 Mb in size [53] in sharp contrast with the housekeeping genes present in GC-rich isochores and tissue-specific genes present in GC-poor isochores, that are 10–50 Kb in size. This contrast was also seen in the large number of GC-poor and GC-rich genes explored recently (Bettecken, T., Moore, A. and Bernardi, G. to be submitted), pointing to two structural classes of genes characterized by different encodings of amino acids and very different intron sizes.

The bimodality of gene distribution was followed by the discovery [38], confirmed and extended by Gilbert et al. (2004) [54], Federico et al. (2006) [55] and Dekker (2007) [56], that the genome core and the genome desert corresponded to open and closed chromatin and were centrally and peripherally located, respectively, in the interphase nucleus of vertebrates (Figure 1C; see Supplementary Table S1 for the properties of gene spaces). In fact, the open and closed chromatin corresponded to euchromatin and heterochromatin, two well-known structures from classical microscopy [57].

As already mentioned, at the evolutionary transition between cold- and warm-blooded vertebrates, the open chromatin of the genome core underwent a GC increase to be stabilized thermodynamically at the higher body temperature of warm-blooded vertebrates (higher GC levels were also observed in 18S RNAs from warm- vs. cold-blooded vertebrates [58]). In contrast, the closed chromatin of the GC-poor genome desert did not evolve towards higher GC levels (see the following section). These observations [45,59] stressed the existence of structural differences at the DNA level between the two gene spaces.

Gene spaces were also clearly detected by mapping DNase-I hypersensitive sites on human isochores [60]. Indeed, hypersensitive site regions (less protected by nucleosomes) are characterized by higher GC levels than the average GC level of the human genome (see Supplementary Figure S1A).

Remarkably, the early apoptotic chicken DNA fragmentation targets a number of specific open chromatin regions with high GC levels, also targeted by micrococcal nuclease digestion with, however, a lower level of GC specificity [61] (see Supplementary Figure S1B).

## 5. The Lamina- and Nucleolus-Associated Domains, Lads and Nads

A new approach, DamID, led to the discovery that genome–lamina interactions occur through more than 1300 sharply defined large domains 0.1–10 Mb in size corresponding to ~35% of the human genome, the Lamina-Associated Domains, LADs, typified by low gene expression levels and demarcated by the insulator protein CTCF, by promoters oriented away from LADs or by CpG islands [62]. In fact, LADs correspond in their properties to GC-poor isochores, that were previously localized at the periphery of the interphase nucleus [38] and also represent ~35% of human DNA. Later investigations [63] showed that LADs were characterized by low gene density, scarcity of CpG islands, high LINEs, low SINEs, and structural conservation across the cold- to warm-blooded vertebrate transition. In contrast, InterLADs showed opposite properties, corresponding to the features of TADs, the Topologically Associated Domains (see the following section, and also Supplementary Table S1). Nucleolus-associated domains, NADs, also exist and show a substantial functional overlap with LADs [64,65].

## 6. Spatial Compartments

Almost 20 years ago, a new approach, Capturing Chromatin Conformation, 3C, was developed to detect the frequency of physical interactions between any pair of genome *loci* [66,67]. The 3C approach was the starting point of an analysis of chromatin structure quite popular in recent years, the Hi-C approach [68], that probes the 3D architecture of the genome by coupling proximity-based ligation with massively parallel sequencing. This approach was claimed to have “identified an additional level of genome organization characterized by the spatial segregation of open, gene-rich, actively transcribed and closed, gene-poor, repressed chromatin to form two genome-wide spatial compartments” that were “arbitrarily” labelled A and B. This statement should, however, be corrected, since the two compartments correspond to the gene spaces (see Figure 1C) that, in fact, had been previously characterized in more detail (see Supplementary Table S1).

The Hi-C approach represented, however, a revolution in the study of chromatin in that (1) it was the first attempt to quantify the proximity of chromatin structures and to use this criterion to define the compartments; (2) it led to the discovery of the short-range spatial organization of genomes into TADs [69,70,71] and of sub-compartments [72]. The Hi-C approach did not provide, however, any information on the DNA present in the compartments, on the evolutionary origins of the latter, nor on molecular explanations concerning the “folding principles of the human genome” [68], namely, the formation of compartments and of TADs, that were later provided by the genomic code.

## 7. Forests and Prairies

The high and low densities of CpG islands of the human genome were used to identify two genomic domains that were called “forests” and “prairies”, respectively [73]. According to the authors, this division partitions the genome into two types of regions that are genetically, epigenetically and transcriptionally different, and “outperform” isochores in the segregation of these properties. These conclusions [74,75] deserve two comments.

The first comment is that forests and prairies confirmed the bimodality of the genome as based on GC levels, gene concentration and DNA structure, and essentially coincide with the “gene spaces” (see Supplementary Table S1). This was expected since CpG islands increase in density with GC levels of isochores [76,77].

The second comment concerns the “outperformance” of forests and prairies compared to separations based on isochores. This point is, in fact, disputable for three reasons: (1) “CpG density distribution can vary greatly among species” [73] as previously found by Aissani and Bernardi, 1991 [76]), as well as within the human genome [78], which implies a lack of generality of the forests and prairies model in contrast with the case of isochores; (2) “Forests and prairies show enhanced segregation from each other in development, differentiation and senescence” [73], again implying a lack of generality; (3) “Genomes having high and uniform CpG distribution can be considered as consisting of mainly forests with little mosaicity”; this conclusion is incorrect as shown by the fact that one of these genomes, the *Drosophila* genome, shows (like some other invertebrate genomes) both high CpG levels and isochores that clearly belong in three families [79].

## 8. Genome Compartments and Isochore “Super-Families”

**Figure 2:**
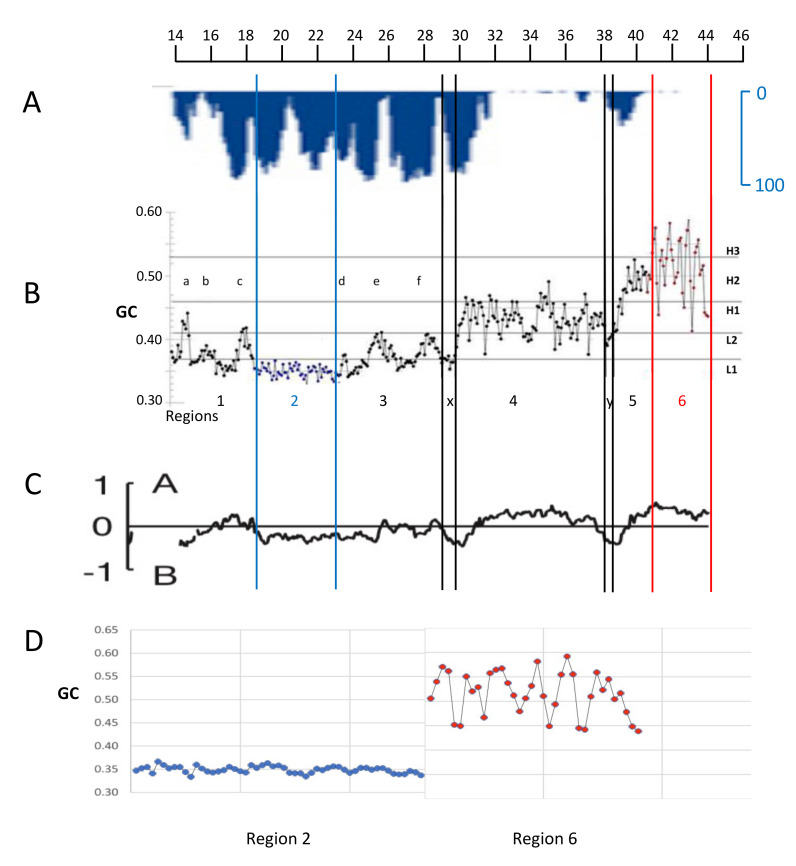
(**A**) The inverted LAD profile of the long arm of human chromosome 21 (from [81]) is compared with the 100 Kb dot-plot GC profile of Figure 3B (from [7]). LADs correspond to L1 isochores (two blue lines bracket region 2, the largest L1 isochore) and to two “valley” isochores (double black lines, X and Y, that belong in the L2^-^ sub-family, a low heterogeneity subfamily in the L2 isochore GC range). One H1 (a), and five L2^+^ single peak isochores (b–f) correspond to InterLADs (the L2^+^ sub-family is a heterogeneous sub-family in the L2 isochore GC range). (**C**) The 100 Kb profile is compared with the A and B compartments (adapted from [82]; the thickness of the profile is due to the enlargement of the original minute figure). Imperfect alignments of Figures (**A**–**C**) are due to the different original sources of the panels. (**D**) The enlarged GC profiles of regions 2 and 6 (corresponding to the telomere) show the striking differences in their compositional ranges (see text).

**Figure 3:**
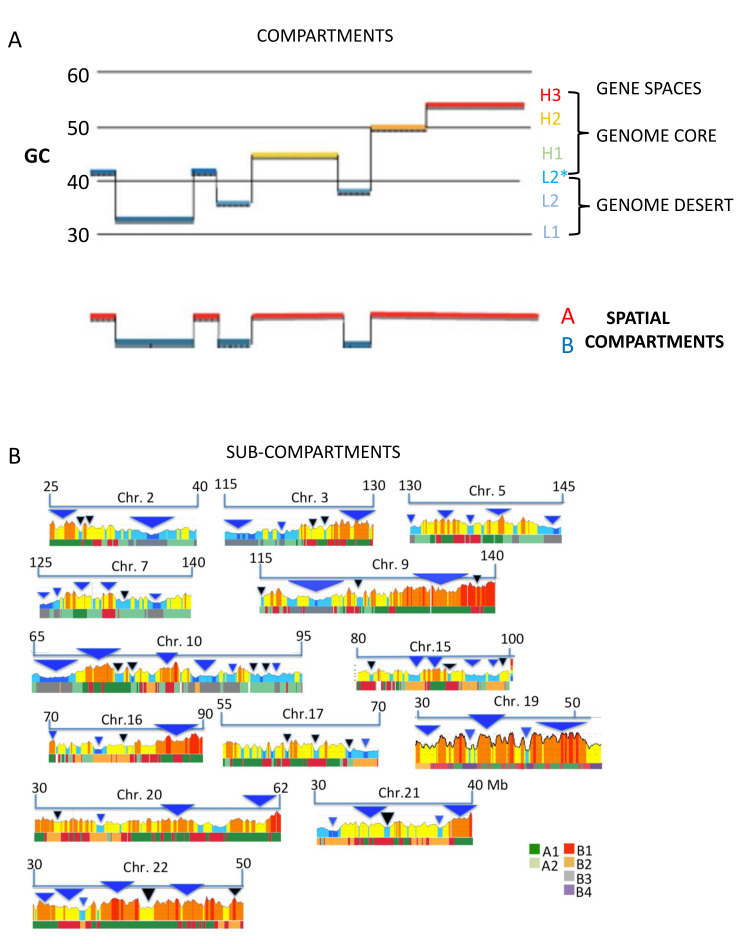
(**A**) A profile of a chromosome segment in which all isochore families are represented is compared with the corresponding compartment profile to show that, while the L1 and L2^−^ isochores correspond to the B compartment, the other isochores correspond to the A compartments (modified from [6]). (**B**) The figure shows an average match between isochores/isochore-blocks (profiles) and chromatin sub-compartments (A1–B4). Blue/black triangles are examples of good/bad matches (from a Supplementary Figure of [30]).

A new approach to the study of isochores, a GC profiling of 100 Kb blocks [6], showed that the isochores of the human genome group are placed into two “super-families” characterized by two long-range 3D structures [8] (see Figure 2A,B and Figure 3A): (1) the GC-poor, low compositional heterogeneity isochores of the L1 family and of the L2^−^ sub-family, both of which correspond to LADs (as well as to the “genome desert”); and (2) the GC-rich isochores that comprise the single- and the multi-peak isochores of the H1–H3 families, and of the L2^+^ sub-family, that correspond to InterLADs, and to the “genome core”.

In human chromosome 21, the first, compositionally flat structure is found in regions 2, X and Y. In contrast, the second one comprises the single peaks and the sets of peaks and valleys of regions 1, 3, 4, 5, 6 (see Figure 2A,B; similar situations are present in all human chromosomes; Jabbari, K., Ritucci, M., Bernardi, G., to be submitted). That the two compartments are “spatially arranged in a polarized manner” in chromosomes was also independently shown by Wang et al. (2016) [80]. Figure 2C shows that the two “super-families” of isochores correspond to the “spatial compartments”, as expected from the comparison of Figure 1C.

Finally, Figure 2D presents an enlargement of two typical regions, 2 and 6, better showing the sharp structural contrast between them, the former covering a ~3% GC range centered at ~35% GC, the latter a ~15% GC range with a lowest level at ~45% GC. In conclusion, the results of Figure 2 not only establish a precise link between the 3D DNA structures of isochore “super-families” and the genome compartments, but also show the existence of different 3D structures in the two different isochore “super-families”.

## 9. Genome Sub-Compartments and Isochores

**Figure 4:**
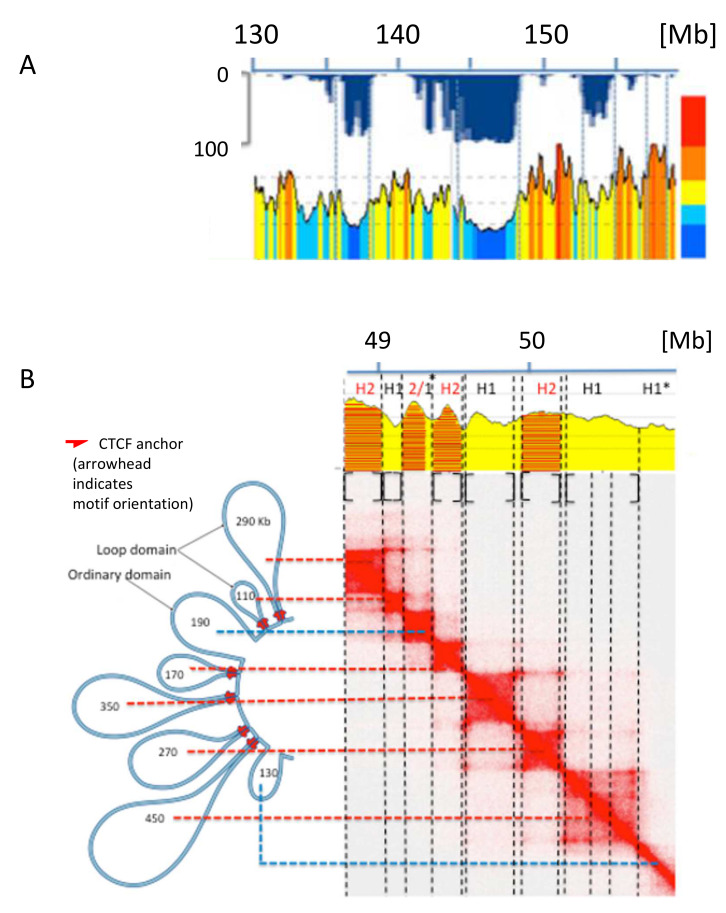
(**A**) ~30 Mb of human chromosome 7 is analyzed at a resolution of 50 Kb; a fine correspondence of isochore boundaries with LAD and TAD boundaries can be observed (modified from [30]). (**B**) The chromatin loops from a 2.1 Mb region of human chromosome 20 have been aligned with the corresponding heat map which was used to segment the corresponding DNA sequence into isochores; asterisks indicate small anomalies in the isochores/domains correspondence (modified from [30]).

Several lines of evidence indicated the existence of finer correlations of isochores (1) with the “sub-compartments” A1, A2 and B1–B3 based on Hi-C [72] that showed links with gene expression and histone modifications; in this case, A1 sub-compartments were shown to correspond to H2/H3 isochores, A2 sub-compartments to H1 and L2^+^ isochores, and B1–B3 sub-compartments to L2^-^ and L1 isochores (see Figure 3B [30]); (2) with LADs and TADs (see Figure 4A), a result obtained for all human and mouse chromosomes [30]; (3) with loop domains (see Figure 4B) [30,83].

**Figure 5:**
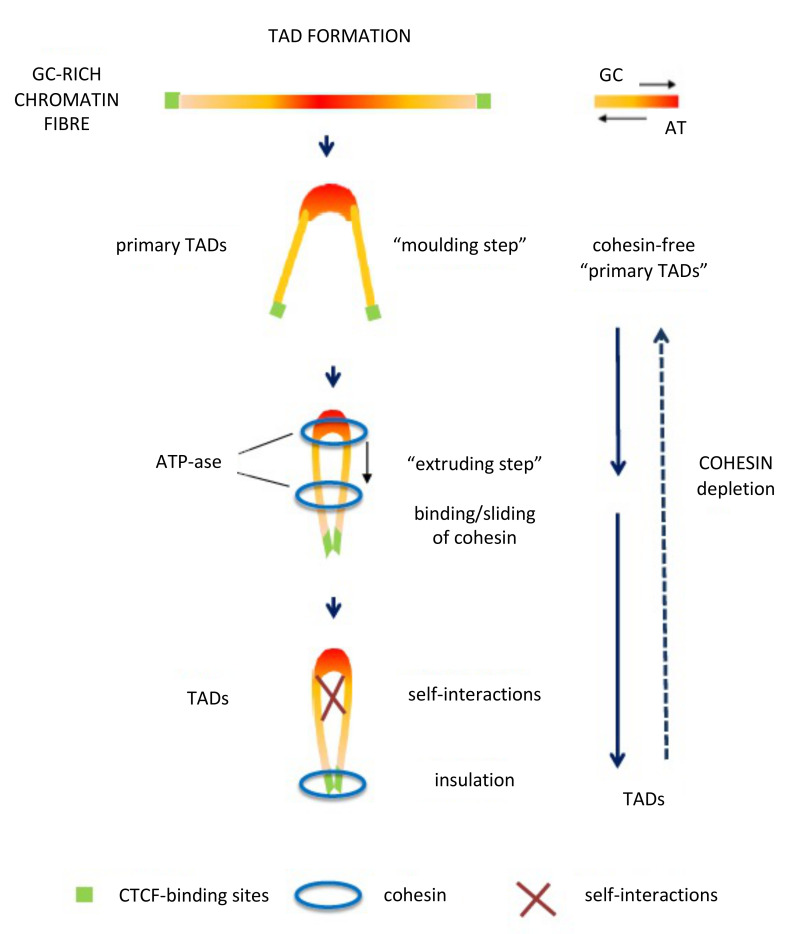
The proposed model for the formation of “primary TADs” and TADs. (Modified from [8]; See text).

In contrast with the compositionally flat B1–B3 sub-compartments, corresponding to L2^−^ and L1 isochore super families, the A1 and A2 sub-compartments corresponding to L2^+^, H1–H3 isochores are characterized by sharp peaks. In this case, compositional gradients (involving oligo-G’s, CpG’s and CpG islands) characterized by increasing bendability, increasing accessibility, decreasing supercoiling and decreasing nucleosome density [84], constrain chromatin into loops, the tips of the loops corresponding to the highest GC levels and being the attachment sites for cohesin, while the increasing presence of oligo-A’s in the valleys helps the folding of the chromatin fibre between GC-rich peaks. This initial “moulding step” leads to cohesin-free “primary TADs” [7,8] that may be followed by extrusion through the action of cohesin, the extrusion being stopped by GC-poor DNA-bound CTCF (see Figure 5). At this point, it should be stressed that the results obtained on a DNA basis and those obtained by Hi-C are linked with each other, as expected.

Extrusion has, however, several problems: (1) removal of cohesin and CTCF from chromosomes showed limited effects on steady-state transcription [85,86,87]; (2) extrusion does not appear to be a general phenomenon in chromatin domains; indeed, it is “malleable and variable between individual cells” [88] and absent in *Drosophila* [89] (in which case building of TADs does not appear to make use of the CTCF/cohesin loop extrusion mechanism) and in many other cases; for instance, the Hi-C approach can fail to detect known structures such as interactions with nuclear bodies, because these DNA regions can be too far apart to directly ligate [90]; (3) extrusion can take place not only through the classical two-sided manner but also through a one-sided manner raising another unsolved problem [91].

Sub-compartments were very recently analyzed by SPIN [92], i.e., by combining compartment mapping and chromatin interaction data. This approach clarified the patterns of specific sub-compartments relative to nuclear speckles, lamina and nucleolus and found specific sequence repeats in sub-compartments.

Another approach to study 3D genome organization and evolution was proposed by Mourad (2019) [93] on the basis of genome sequences only, more specifically by comparing the distances between convergent and divergent CTCF motifs (ratio R) to detect CTCF looping. R values were very high for L1 isochores and sub-compartments B3 (corresponding to heterochromatin) and very low for H3 isochores.

## 10. Short Sequences in Isochores and Nucleosome Binding

**Figure 6:**
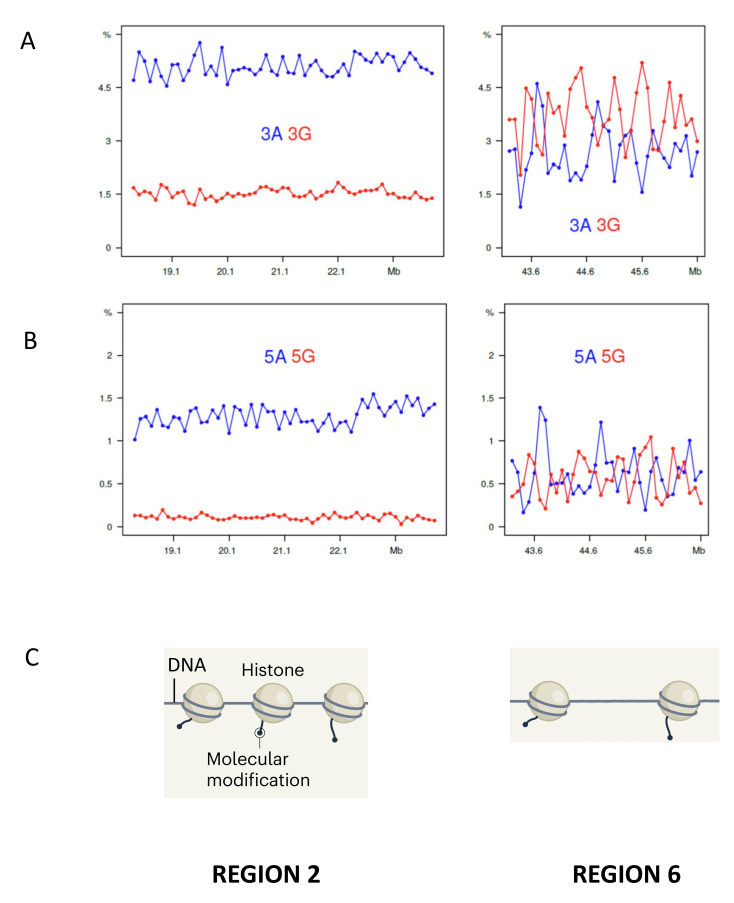
(**A**,**B**) Profiles of tri- and penta- A’s and G’s of regions 2 and 6 (from Figure S3 of [108] (**C**) A scheme of nucleosome densities for regions 2 and 6. (Modified from [109]; See also legend of Figure 7).

Specific short sequences are characteristic of isochore families, as shown by the “short sequence designs” of isochores [15,94] and the following results: (1) Previous investigations [95,96] indicated that specific sets of di- and tri-nucleotides, such as “AAA/TTT” and “GGG/CCC”, as well as the “A/T-only” and “G/C-only” trinucleotides, have widely different distributions in different isochore families of the human genome, the former being predominant in GC-poor, the latter in GC-rich isochores, as expected; (2) Profiles of all 64 trinucleotides of chromosome 21 confirm an essentially bimodal topology with compositionally flat (yet different) levels in GC-poor isochores and single or multiple peaks in GC-rich isochores [9]; (3) Figure 6A,B shows the profiles of oligo-A’s and oligo-G’s of regions 2 and 6 of chromosome 21 that are typical of GC-poor and GC-rich isochores; these results indicate that region 2 shows essentially flat levels for 3G and 5G with small blocks of oligo A’s, whereas region 6 shows sharp peaks of both 3A and 5A, as well as of 3G and 5G.

In conclusion, two different classes of isochores can be distinguished on the basis of short-sequence profiles, an important point because these profiles define the nucleosome binding pattern. Indeed, investigations on DNA/nucleosome interactions have shown (1) that DNA sequences differ greatly in their ability to bend sharply [97,98] and that nucleosome formation is highly dependent on specific DNA sequences [99,100], AT and GC tracts being intrinsically inhibitory [101,102]; and (2) that CpGs and CpG islands that increase in density with GC levels are strongly inhibitory for nucleosome binding [102,103,104]. These observations led to the concept of a “nucleosome positioning code”, interestingly also called “a genomic code for nucleosome positioning” [101].

An important new point is that nucleosome positioning is bimodal, in that it is denser in the regions corresponding to GC-poor isochores that are characterized by the compositionally least heterogeneous L1 and L2^−^ isochores. In this case, larger and deeper “clutches” of nucleosomes correspond to the “closed” heterochromatin, whereas “open chromatin” is formed by smaller and less dense clutches that associate with RNA Polymerase II [105]. Indeed, the internucleosome sequences are increasingly broader with increasing GC and increasing amounts of sequences (like CpGs and CpG islands) that are incompatible with nucleosome binding. Finally, there is evidence that nucleosomes remember where they were [106]. In other words, there are two sets of nucleosome spacings, a short one and a long one (see Figure 6C and Figure S1A,B).

Interestingly, the trinucleotide patterns in the 0.1–0.5 Kb sequences flanking genes located in GC-rich and GC-poor isochores indicate differences in the transcription factors that bind upstream and downstream of genes [96]. This result indicates differences in the regulation of genes located in different isochore families, in agreement with the fact that different classes of genes are located in different isochore families [107].

## 11. Conclusions

### 11.1. The Correlations of DNA Sequences with Chromatin Architecture

These correlations were investigated at three DNA size levels (see Supplementary Table S2). The long-range bimodal compartmentalization of chromatin was shown to be associated with the different DNA structures of the two “super-families” of isochores, a compositionally flat one, and a single peak or peak-and-valley one. The demonstration of correlations between the 3D structure of isochore “super-families” and compartments is important, if one considers that it is currently admitted that the “exact molecular players that drive compartment organization are not yet known” [108] and that “the molecular determinants that modulate the maintenance and movement of compartmentalization remain largely elusive” [92] or are due to “self-associating homeotypic chromatin types” [88]. In fact, “attractions between heterochromatin regions were proposed to be essential for the phase separation of the active and inactive genomes”, and “interactions of the chromatin with the lamina are necessary to build the conventional architecture from these segregated phases” [110] (Falk et al. 2019). The interaction of GC-poor sequences from both cold- and warm-blooded vertebrates with lamina provides support to this proposal.

At a short-range resolution, “primary TADs” [7,8,9] appear to be constrained into loops by the properties that accompany the GC gradients of isochore peaks, to which they correspond, namely, increasing bendability, increasing nuclease accessibility, decreasing supercoiling and decreasing nucleosome density. This is again an important conclusion, if one considers that extrusion was visualized as the only way to explain the formation of the loops [108,111], a view also contradicted by the fact that extrusion may or may not affect “primary TADs”. Two important results concern the correspondence of isochores and sub-compartments with LADs and TADs (Figure 4A) and with loop domains (Figure 4B). The compositional stability of the GC-poor isochores of LADs across the evolutionary transition between cold- and warm-blooded vertebrates are most likely to be due to their interactions with the lamina as already mentioned. In contrast, the isochores that are not interacting with lamina underwent a GC increase (and an increase in compositional heterogeneity) in order to be stabilized at the higher body temperature of warm-blooded vertebrates. Needless to say, the correlations of DNA sequences with genome organization explain why isochores are “a fundamental level of genome organization” [112], even if when this was proposed on the basis of our work, it was not clear what the real reason was.

Finally, at the short sequence level, the two sets of sequences, GC-rich and GC-poor, that characterize the compartments and sub-compartments define nucleosome positioning. It is understandable that the thermal transition is accompanied by a decreased density of nucleosomes, because of the increasing amounts of GC-rich sequences (such as CpG and CpG islands) that are incompatible with nucleosome binding and that are located in the internucleosome linkers.

It should be stressed that the structure of DNA sequences and not nucleosome distribution is primarily responsible for moulding chromatin architecture. Indeed, DNA structure not only exists independently of nucleosome binding, but it is responsible for it. This is shown by the striking finding that TADs are not very different in fibroblasts and in spermatozoa [113], in which latter case nucleosomes are replaced by protamines (see Supplementary Figure S2).

### 11.2. The Genomic Code

The properties of DNA sequences, as seen at the levels just discussed, mould chromatin architectures according to a “genomic code” [6,7,8] coding sequences and the regulatory sequences (see Table 1). The importance of the sequences under discussion is underlined by the fact that they pervade the totality of the genome (including satellite DNAs; see Figure S3), overlapping and compositionally constraining not only transposons and long non-coding RNAs, but also the coding sequences. Indeed, the composition of codons for amino acids varies according to the composition of the corresponding isochores.

The genomic code defines two vast ensembles in the vertebrate genome. The first is characterized by the GC-poorest, least heterogeneous sequences and represents ~35% of the genome, that are associated with the lamina, have a high nucleosome density, and are compositionally stable through the cold- to warm-blooded vertebrates transition. The second one, corresponding to the GC-rich sequences, ~65% of the genome, is characterized by increasing compositional heterogeneity with increasing number of sequences that are incompatible with nucleosome binding, leading, as a consequence, to a wider internucleosome spacing.

It should be stressed that the approach used in the experiments presented, based as it is on the role of DNA sequences in the moulding of chromatin architecture, is totally different from the strategy prevailing over the last few years in the field of chromatin, a strategy which is based on the proximity of chromatin structures. It is not surprising, therefore, that while the latter strategy discovered sub-compartments and TADs, it could not provide explanations at the molecular level for the “folding principles of the human genome” [68] that could be obtained by using DNA sequences.

One can now ask whether any of the properties of the genomic code were reported before: (1) Trifonov (1980) [114] remarkably predicted the sequence-dependent deformational anisotropy of chromatin DNA, as well as the possibility of overlapping codes, one of which, the “chromatin code”, would provide instructions on appropriate placement of nucleosomes along DNA molecules and their spatial arrangement [115]; (2) the work of Todolli et al. (2017) [116] pointed to local sequence-dependent features found in high-resolution structures that introduce irregularities in the disposition of adjacent residues that facilitate the specific binding of proteins and also determine the positions of nucleosomes on DNA and the lengths of the interspersed DNA linkers; (3) Ramirez et al. (2018) [117] identified eight DNA motifs enriched at TAD boundaries and proposed that DNA sequence guides the genome architecture by allocation of boundary proteins in the genome; (4) Gorkin et al. (2018) [118] identified thousands of regions in the limphoblastoid cell lines from 20 individuals where 3D chromatin varies and demonstrated that common DNA sequence variants can influence chromatin conformation, a result expected from the moulding of chromatin by DNA sequences [6,7,8,9]; (5) Fudenberg et al. (2020) [119] and Schwessinger et al. (2020) [120] developed methods that predict genome folding from DNA sequences alone. In other words, the old predictions of Trifonov and recent results are in agreement with the present conclusions.

At this point, three remarks should be done. (1) Satellite DNA sequences (see Supplementary Figure S3 for an example) appear at precise locations; (2) Non-B-form DNA is enriched at centromeres [121]; (3) Transposons and long-non-coding RNAs are expressed while overlapping DNA.

### 11.3. The Genomic Code and “Junk DNA”

When it was realized that the coding sequences represented a small percentage of the human genome, it was proposed that the majority of it was “junk DNA” [5]. This was the beginning of the longest debate in molecular evolution, which is still going on after almost 50 years [122,123,124,125,126], justified as it was by the lack of alternative explanations. The discovery of the moulding of chromatin structure by DNA sequences, the genomic code, leads in fact to an explanation (see Table 1) which is alternative to the idea that “perhaps 90% of our DNA, though biochemically active, does not contribute to fitness in any sequence-dependent way and possibly in no way at all” [126]. Indeed, the sequences involved in moulding chromatin (and contributing to fitness) occupy the totality of genome DNA leaving no room for “junk DNA”. It should be stressed, however, that while single nucleotide changes in the sequences that are the backbones of chromatin are unlikely to lead to structural variations being neutral [127,128] or nearly neutral [129,130], short-sequence deletions/insertions, can lead to somatic structural variations such as the fusion of TADs and complex rearrangements that markedly change chromatin folding maps in the cancer genomes [131] and most probably in aging and a number of diseases.

### 11.4. The Genomic Code and ENCODE

**Figure 7:**
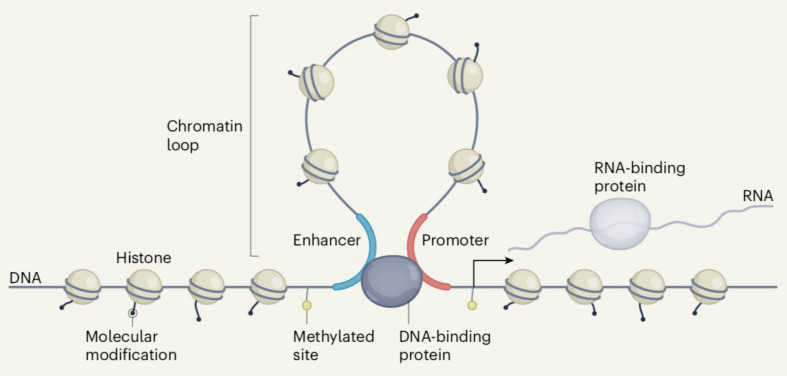
Functional elements across the human genome [109]. This Figure can also be seen as a model representing the structural elements discussed in the article (see text). This figure is published with the permission of authors and Springer Nature.

At this point, it is of interest to compare the views of the human genome as derived from the structural approach discussed so far and from ENCODE, the Encyclopedia of DNA Elements, which is focused on the functional properties of the genome. Figure 7 presents the functional elements as derived from ENCODE [109]. This concept is of great interest because it can be also read as representing the picture derived from the structural work discussed here. Indeed, there are two major structural elements, described so far, that appear in the figure. The horizontal bottom line of nucleosomes can be seen as corresponding to LADs, whereas the loop corresponds to a TAD. (Incidentally, the nucleosome spacing correctly happens to be larger in the latter case compared to the former one). In other words, the Figure can be read not only as presenting the functional elements but also the structural elements. In other words, the results of our structural approach can be seen as matching the results of the functional approach of ENCODE.

## Figures and Tables

**Table 1:** The four pillars of the genome ^(a)^.

	THE DOUBLE HELIX		
REGULATORYSEQUENCES	TRANSPOSONS	LONG NON-	CODING SEQUENCES
		CODING RNAs	
	***JUNK DNA***		
		THE GENOMIC CODE		

^(a)^ Titles in the diagram: Black: The original three pillars. The double helix fills the totality of the genome DNA. The coding sequences correspond to less than 2% of the genome. The regulatory sequences also correspond to a minute part of the genome. Blue: The “junk DNA” fills in the space not occupied by the coding and regulatory sequences. Its existence is ruled out by the genomic code. Green: Transposons and long non-coding RNAs. Red: The genomic code, the fourth pillar, is pervasive and fills the totality of the genome.

## Data Availability

Not applicable.

## Supplementary Materials

The following are available online at https://www.mdpi.com/article/10.3390/life11040342/s1, Table S1: Human Genome Compartmentalization, Table S2: The Bimodal Organization of The Human Genome at Three DNA Size Levels, Figure S1: A. Compositional distribution of human DNA and of hypersensitive sites, Figure S2: TADs as present in sperm cells and fibroblasts of a region of chromosome 19, Figure S3: Compositional profile of the short arm and of the centromere (α-satellite) of chromosome 21 (by Gregorio Bernardi).
