# Supplementary material for: The “Genomic Code”: DNA Pervasively Moulds Chromatin Structures Leaving no Room for “Junk”
Source: Life (Basel). 2021 Apr 13;11(4):342. doi: 10.3390/life11040342 (PMC8070607; doi:10.3390/life11040342)
Supplement: Supplementary file 1 [file life-11-00342-s001.pdf]

life

MDPI

*Supplementary Materials*

# The "Genomic Code": DNA Pervasively Moulds Chromatin Structures Leaving No Room For "Junk"

Table S1. Human Genome Compartmentalization.

| **GENE SPACES**[1–4] | **InterLADs**<br>**LADs** [5,6] | **SPATIAL COMPARTMENTS**<br>[7,8] | **FORESTS, PRAIRIES**<br>[9,10] |
|---|---|---|---|
| **The genome core**<br>GC-rich<br>Gene-rich<br>Open chromatin<br>Central location in the interphase nucleus<br>(Euchromatin)<br>High gene expression<br>Small introns, small UTR<br>High GC heterogeneity<br>High CpG islands<br>High SINEs<br>Low LINEs<br>Early replication timing<br>High recombination | **InterLADs** | **A compartment** [7,8]<br>(GC-rich) [7]<br>Gene-rich<br>Open chromatin<br>Actively transcribed<br>Proximity ligation | **Forests**<br>CpG islands-rich |
| **The genome desert**<br>GC-poor<br>Gene-poor<br>Closed chromatin<br>Peripheral location in the interphase nucleus<br>(Heterochromatin)<br>Low gene expression<br>Large introns, long UTR<br>Low GC heterogeneity<br>Low CpG islands<br>Low SINEs<br>High LINEs<br>Late replication timing<br>Low recombination | **LADs** [5,6]<br>GC-poor<br>Gene-poor<br>Low CpG islands<br>Low SINES<br>High LINES<br>Low H3K4me2 | **B compartment** [7,8]<br>(GC-poor) [7]<br>Gene-poor<br>Closed chromatin<br>Repressed chromatin<br>Proximity ligation | **Prairies**<br>CpG islands-poor |

**Table S2.** The Bimodal Organization of The Human Genome at Three DNA Size Levels.

| | |
|---|---|
| **COMPARTMENTS/ GENE SPACES** | |
| **Genome core** | Genome desert |
| InterLADs | LADs |
| Compartment A | Compartment B |
| Forests | Prairies |
| GC-rich*<br>Isochore super-families:<br>$L2^+$ sub-family<br>H1, H2, H3 families | GC-poor*<br>Isochore super-families:<br>L1 family<br>$L2^-$ sub-family |
| **SUB-COMPARTMENTS/ DOMAINS** | |
| A1 H2,H3<br>A2 H1,$L2^+$<br>TADs | B1-B3 $L2^-$,L1<br>LADs** |
| GC-rich isochores | GC-poor isochores |
| **SHORT SEQUENCES** | |
| GC-rich | GC-poor |

*Red characters : DNA properties, **LADs may also be classified as compartments.

## Supplementary Figure Legends

A

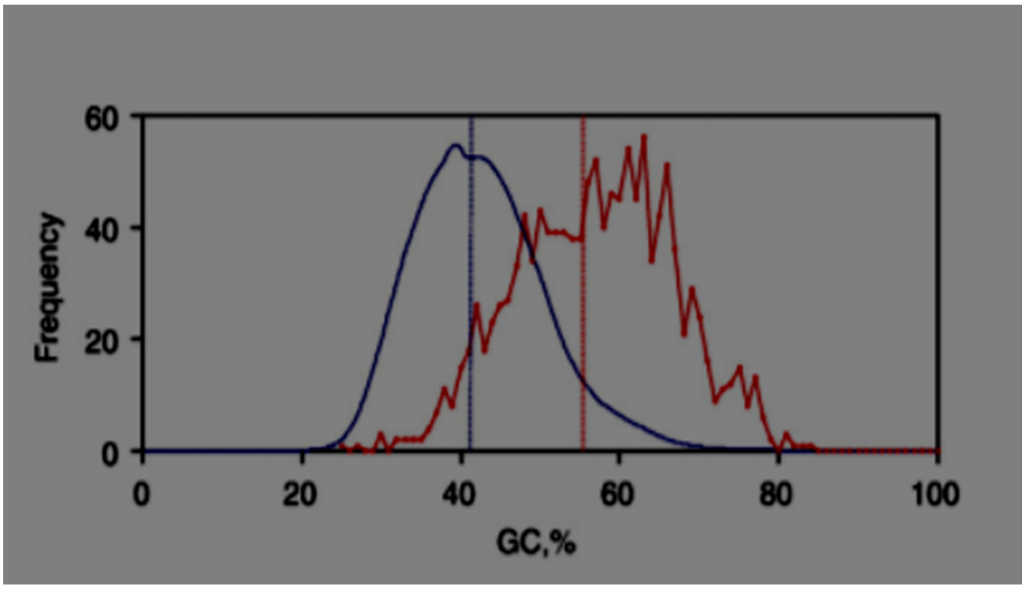


B

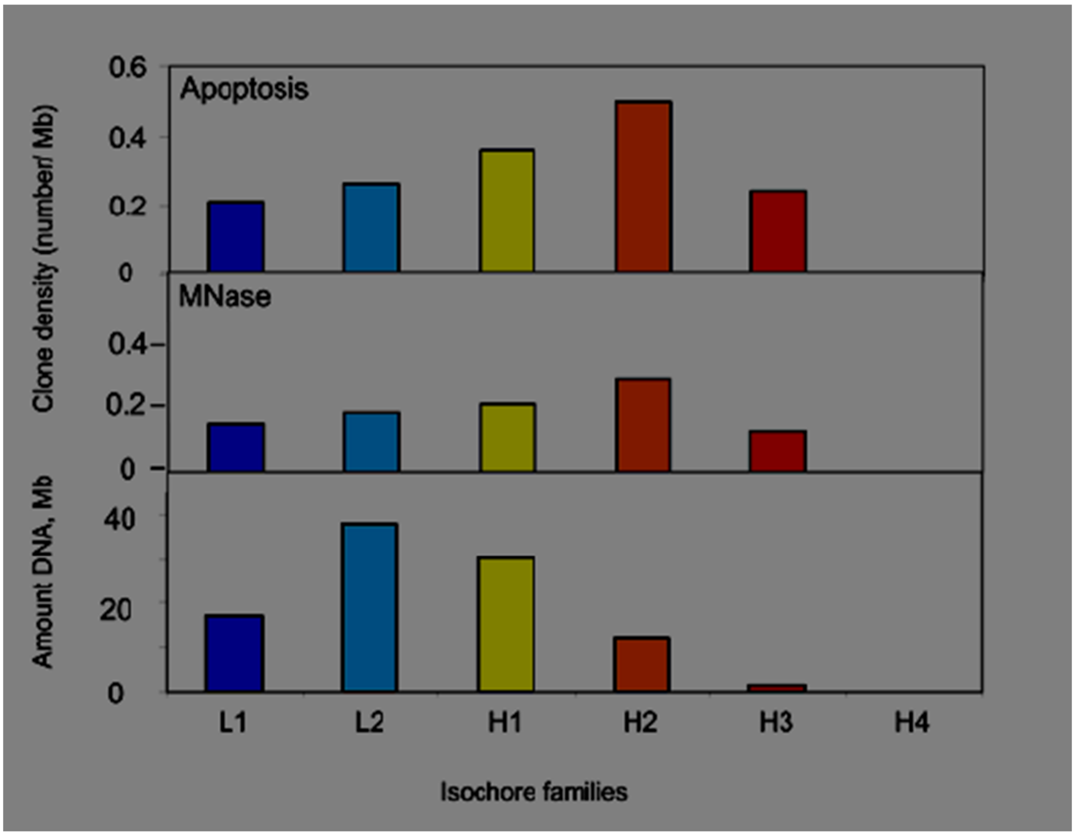


Figure S1. **A.** Compositional distribution of human DNA and of hypersensitive sites. The sites are extremely sensitive to enzymatic digestion by DNase-1 compared to the surrounding sequences because they are poorly protected by histones. The GC profile of the sequences (average length ~500 bp) corresponding to hypersensitive sites (red line), downloaded from http:// research.nhgri.nih.gov/DNaseHS/chip_2006, was superimposed on the GC profile of the human genome (in blue), as calculated at a window size of 500 bp. The average GC level of the sequences analysed was found to be ~56% (red line), whereas that of the human genome is ~41% (blue line). [6]. **B.** Density of apoptotic and micrococcal nuclease (MNase), Low Molecular Weight (LMW) cloned DNA fragments in isochore families of the chicken genome. The bottom panel shows the amount of DNA in each isochore family. [7]

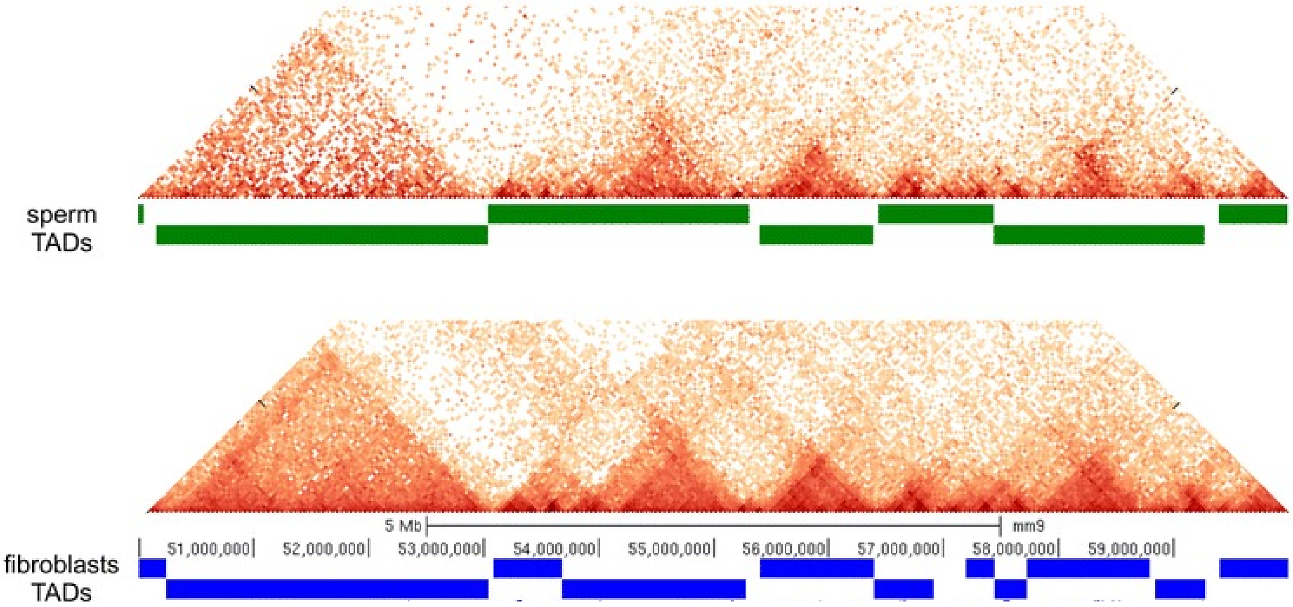


**Figure S2.** TADs as present in sperm cells and fibroblasts of a region of chromosome 19. The TAD signal shows visible similarity between sperm cells and fibroblasts in spite of the fact that the nucleosomes of TADs are replaced by protamines in sperm cells. [1].

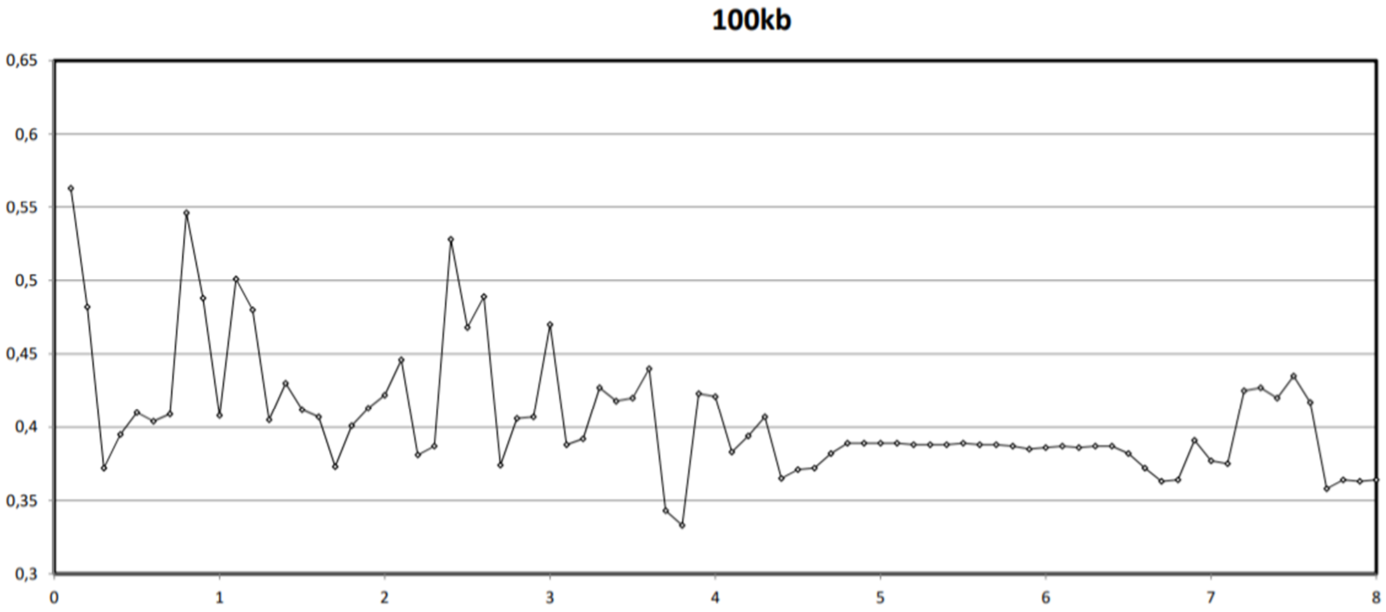


**Figure S3.** Compositional profile of the short arm and of the centromere ($\alpha$-satellite) of chromosome 21 (by Gregorio Bernardi).

## References


1. Battulin, N.; Fishman, V.S.; Mazur, A.M.; Pomaznoy, M.; A Khabarova, A.; A Afonnikov, D.; Prokhortchouk, E.B.; Serov, O.L. Comparison of the three-dimensional organization of sperm and fibroblast genomes using the Hi-C approach. Genome Bi-ol. **2015**, *16*, 1–15
2. Bernardi, G. Isochores and the evolutionary genomics of vertebrates. Gene **2000**, *241*, 3–17
3. Bernardi, G. The human genome: organization and evolutionary history. Annu. Rev. Genet. **1995**, *29*, 445-476
4. Bernardi, G. The vertebrate genome: isochores and evolution. Mol. Biol. Evol. **1993**, *10*, 186–204
5. Dekker, J.; Rippe, K.; Dekker, M.; Kleckner, N. Capturing Chromosome Conformation. Sci. **2002**, *295*, 1306–1311
6. Di Filippo, M.; Bernardi, G. Mapping DNase-I hypersensitive sites on human isochores. Gene **2008**, *419*, 62–65.
7. Di Filippo, M.; Bernardi, G. The Early Apoptotic DNA Fragmentation Targets a Small Number of Specific Open Chromatin Re-gions. PLOS ONE **2009**, *4*, e5010

8. Guelen, L.; Pagie, L.; Brasset, E.; Meuleman, W.; Faza, M.B.; Talhout, W.; Eussen, B.H.; De Klein, A.; A Wessels, L.F.; De Laat, W.; et al. Domain organization of human chromosomes revealed by mapping of nuclear lamina interactions. Nat. Cell Biol. **2008**, *453*, 948–951
9. Lieberman-Aiden, E.; Van Berkum, N.L.; Williams, L.; Imakaev, M.; Ragoczy, T.; Telling, A.; Amit, I.; Lajoie, B.R.; Sabo, P.J.; Dorschner, M.O.; et al. Comprehensive Mapping of Long-Range Interactions Reveals Folding Principles of the Human Genome. Science **2009**, *326*, 289–293
10. Liu, J. From 1D sequence to 3D chromatin dynamics and cellular functions: a phase separation perspective. *Nucl. Ac. Res.* **2018**, *46*, 9367-9383
11. Meuleman, W.; Peric-Hupkes, D.; Kind, J.; Beaudry, J.-B.; Pagie, L.; Kellis, M.; Reinders, M.; Wessels, L.; Van Steensel, B. Constitutive nuclear lamina-genome interactions are highly conserved and associated with A/T-rich sequence. Genome Res. **2012**, *23*, 270–280.
12. Quan, H.; Yang, Y.; Liu, S.; Tian, H.; Xue, Y.; Gao, Y.Q. Chromatin structure changes during various processes from a DNA sequence view. Curr. Opin. Struct. Biol. **2020**, *62*, 1–8.
13. Saccone, S.; Federico, C.; Bernardi, G. Localization of the gene-richest and the gene-poorest isochores in the interphase nuclei of mammals and birds. Gene **2002**, *300*, 169–178.
